# Supplementary material for: Polarity of the CRISPR roadblock to transcription
Source: Nat Struct Mol Biol. 2022 Dec 5;29(12):1217–27. doi: 10.1038/s41594-022-00864-x (PMC9758054; doi:10.1038/s41594-022-00864-x)
Supplement: Supplementary file 1 — Supplementary Tables 1–5. [file 41594_2022_864_MOESM1_ESM.pdf]

nature structural & molecular biology

Article https://doi.org/10.1038/s41594-022-00864-x

Polarity of the CRISPR roadblock to transcription

In the format provided by the authors and unedited

Table of Contents:

- Supplementary Table 1
- Supplementary Table 2
- Supplementary Table 3
- Supplementary Table 4
- Supplementary Table 5

Category	dCas9 PAM Distal	dCas9 PAM Proximal
Before Chase		
$F_{\mathrm{A20,dCas\_i}}$	0.91	0.93
$F_{\mathrm{A20\_only\_i}}$	0.05	0.07
$F_{\mathrm{dCas\_only\_i}}$	0.05	0.00
$F_{\mathrm{Nak\_i}}$	0.00	0.00
Total	1.00	1.00
After Chase		
$F_{\mathrm{TEC\_up,dCas\_f}}$	0.29	0.32
$F_{\mathrm{TEC\_up\_only\ \_f}}$	0.03	0.05
$F_{\mathrm{dCas\_only\ \_f}}$	0.03	0.05
$F_{\mathrm{Coll\_f}}$	0.29	0.54
$F_{\mathrm{dCas\_rem\_f}}$	0.06	0.00
$F_{\mathrm{TEC\_dn\ \_f}}$	0.26	0.02
$F_{\mathrm{Nak\_f}}$	0.03	0.02
Total	1.00	1.00

Calculated Results		
	dCas9 PAM Distal	dCas9 PAM Proximal
$P_{\mathrm{Coll\_Comp}}$	0.64	0.61
$P_{\mathrm{Read-through}}$	0.48	0.00
P_{Removal}	0.58	0.00

Supplementary Table 1. Trace categories for assaying transcription read-through of a bound dCas protein.

This tables shows the detailed trace category classification for RNAP approaching a bound dCas9 complexed with an unmodified RNA from two representative sample chambers, one for PAM-distal and one for PAM-proximal. These fractions (top) are used to compute various probabilities (bottom).

gRNA	RNA sequence (5' -> 3')
Cas9 unmodified gRNA	GCGCGUAUCAUCCCUUACCG + 80 bp (crRNA + tracrRNA)
Cas9 **3-nt mismatch** gRNA<br>Cas9 **5-nt inverted repeat** gRNA<br>Cas9 **6-nt inverted repeat** gRNA<br>Cas9 **7-nt inverted repeat** gRNA<br>Cas9 **6-nt non complementary** gRNA	CGCCGUAUCAUCCCUUACCG + 80 bp (crRNA + tracrRNA)<br>CGCGCUGCGCGUAUCAUCCCUUACCG + 80 bp (crRNA + tracrRNA)<br>ACGCGCUGCGCGUAUCAUCCCUUACCG + 80 bp (crRNA + tracrRNA)<br>UACGCGCUGCGCGUAUCAUCCCUUACCG + 80 bp (crRNA + tracrRNA)<br>AUAUUGGCGCGUAUCAUCCCUUACCG + 80 bp (crRNA + tracrRNA)
Cas12a unmodified gRNA	GGUAAUUUCUACUCUUGUAGAUGCCAUUCCCUACUAUGCGCG

Supplementary Table 2. gRNAs used in this study.

Custom Cas9 sgRNAs were purchased from Sigma-Aldrich. Cas12a gRNAs were made by *in vitro* transcription as described in Methods.

Oligo Name	Applications	Sequence (5’ -> 3’)
pUC19_AsC12a_gRNA_Temp_KLD_F<br><br>pUC19_AsC12a_gRNA_Temp_KLD_R	Generate pUC19 with T7 transcription construct for As-Cas12a to generate gRNA.	ACTATGCGCGGGGCGGCATGGTCCCAGC<br><br>AGGGAATGGCATCTACAAGAGTAGAAATTACCTATAGTGAGTCGTATTAGAATTCACTG
PH_pUC19_PCR_Temp_F<br><br>PH_pUC19_PCR_Temp_R	Generate a template via PCR to perform T7 transcription and generate As-Cas12a guide RNA.	CAGGGTTTTCCCAGTCACGA<br><br>CATTAGGCACCCCAGGCTTT
3.1kb_Temp_F_DraIII<br><br>3.1kb_Temp_R	Generate co-directional unzipping trunks from different plasmids via PCR.	GACTCACCTAGTGTGAATTCGAGCTCGGTACC<br><br>CAGTTCGTCATACTGCTCAGCCAG
1.1 kb_Rev_Temp_F<br><br>1.1 kb_Rev_Temp_R_AlWNI	Generate the inverted unzipping templates via PCR for mapping PAM-proximal dCas and downstream TEC.	TGAATTCGAGCTCGGTACC<br><br>GACTCAGCTACTGCTTTACCTGCGATGTACTCAAC
pRL574_C9_NTS_Flank_KLD_F<br><br>pRL574_C9_NTS_Flank_KLD_R	Generate the PAM proximal Cas9 target region on pRL574 via site directed mutagenesis.	ATGATACGCGCGTTATACAGCACTTTACCCGCGATCCGAAGGACAACCT<br><br>CCCTTACCGTGGTTCCTGGCTGGACTTCGAATAAGAGTGGGTTTTACCTTTG
pRL574_C12a_NTS_Flank_KLD_F<br><br>pRL574_C12a_NTS_Flank_KLD_R	Generate the PAM-distal Cas12a target region on pRL574 via site directed mutagenesis.	GGAATGGCGAAATTCCTGGCTGGACTTCGA<br><br>CTACTATGCGCGGTTATACAGCACTTTACCCG
pRL574_C12a_TS_Flank_KLD_F<br><br>pRL574_C12a_TS_Flank_KLD_R	Generate the PAM-Proximal Cas12a target region on pRL574 via site directed mutagenesis.	CTACTATGCGCGGTTATACAGCACTTTACCCGTCGATCCGAAGGACAACC<br><br>GGAATGGCGAAATTCCTGGCTGGACTTCGAATAAGAGTGGGTTTTACCTTTG
pBR322_2867bp_yarm_F<br><br>pBR322_2867bp_yarm_R	Generate Arm 1 and Arm 2 of the Y template via PCR	CGCGTTTCGGTGATGACGGTGA<br><br>GTTACGGATCCGCGCTCGGCCCTTCCGG
pmh_hTm_Upper 1<br><br>pmh_hTm_Lower 1p	Adapters to be annealed and ligated to Arm 1	CAGCGCCAGACTGGGGGCGTCCTGCAGAAGGCTCCCACGACGACACCGAC<br><br>pGGGAGTCGGTGTCGTCGTGGGAGCCTTCTGCAGGACGCCCCCAGTCTGGCGCTGGCGGTCCGCTCTACGCACACGCATCTGGGTCTA
pmh_hTm_Upper 2p<br><br>pmh_hTm_Lower 2p	Adapters to be annealed and ligated to Arm 2	pGGGACAGGTGGGGTGCTTACTGAGGAATAAGACGTGACCGCGCCTGTCGCAGAC<br><br>pACCCAGATGCGTGTGCGTAGAGCGGACCGCGTCCACCCCACGAATGACTCCTTATTCTGCACTGGCGCGGACAGCGTCTG
Gel_Temp_5pBio_F	Forward primer to generate templates for transcription gels. Contains a 5’-biotin modification. Used with “1.1 kb_Rev_Temp_R_AlWNI”	\5Biosg\AAAACGACGGCCAGTGAA

Supplementary Table 3. DNA oligonucleotides used in this study.

All DNA oligonucleotides were purchased from Integrated DNA Technologies (IDT). ‘p’ indicates a 5’ phosphate modification to allow ligation.

Name	Diagram	Target Sequence, PAM and Flanking DNA
Transcription collision from Cas9 PAM-distal side	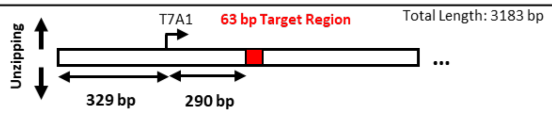	CGGGTAAAGTGCTGTATAACGCGCGTATCATCCCTTA<br>CCGTGGTTCCTGGCTGGACTTCGAAT
Transcription collision from Cas9 PAM-proximal side	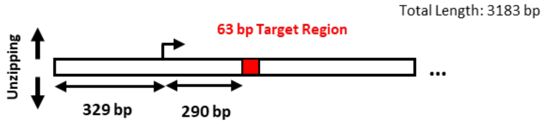	ATTCGAAGTCCAGCCAGGAACCACGGTAAGGGATG<br>ATACGCGCGTTATACAGCACTTTACCCG
Transcription collision from Cas12a PAM-distal side	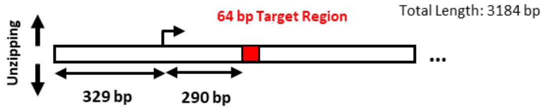	CGGGTAAAGTGCTGTATAACCGCGCATAGTAGGGAA<br>TGGCGAAATTCCTGGCTGGACTTCGAAT
Transcription collision from Cas12a PAM-proximal side	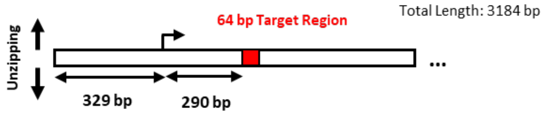	ATTCGAAGTCCAGCCAGGAATTTCGCCATTCCCTACT<br>ATGCGCGGTTATACAGCACTTTACCCG
Cas9 PAM proximal mapping	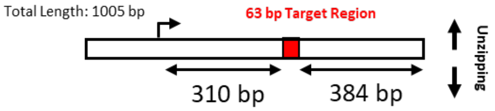	ATTCGAAGTCCAGCCAGGAACCACGGTAAGGGATG<br>ATACGCGCGTTATACAGCACTTTACCCG
Cas12a PAM-proximal mapping	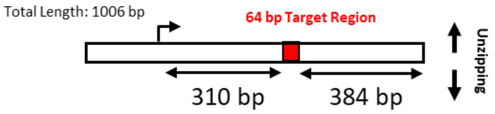	ATTCGAAGTCCAGCCAGGAATTTCGCCATTCCCTACT<br>ATGCGCGGTTATACAGCACTTTACCCG

Supplementary Table 4. Unzipping templates used in this study.

For each template, we indicate its overall features.

Name	Diagram
Transcription collision from Cas9 PAM-distal side	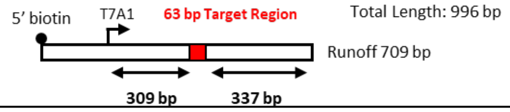
Transcription collision from Cas9 PAM-proximal side	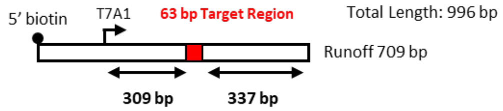
Transcription collision from Cas12a PAM-distal side	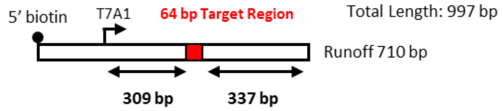
Transcription collision from Cas12a PAM-proximal side	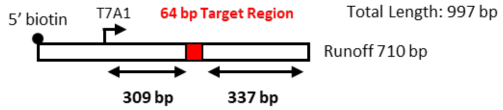

Supplementary Table 5. Transcription gel templates used in this Supplementary Fig. 6.

For each biotinylated template used in the bulk transcription, we indicate its overall features.
