# Supplementary material for: Polarity of the CRISPR roadblock to transcription
Source: Nat Struct Mol Biol. 2022 Dec 5;29(12):1217–27. doi: 10.1038/s41594-022-00864-x (PMC9758054; doi:10.1038/s41594-022-00864-x)
Supplement: Supplementary file 2 — Reporting Summary [file 41594_2022_864_MOESM2_ESM.pdf]

nature portfolio

Corresponding author(s): Michelle D. Wang

Last updated by author(s): 9/30/2022

Reporting Summary

Nature Portfolio wishes to improve the reproducibility of the work that we publish. This form provides structure for consistency and transparency in reporting. For further information on Nature Portfolio policies, see our Editorial Policies and the Editorial Policy Checklist.

Statistics

For all statistical analyses, confirm that the following items are present in the figure legend, table legend, main text, or Methods section.

n/a	Confirmed	
☐	☒	The exact sample size (n) for each experimental group/condition, given as a discrete number and unit of measurement
☐	☒	A statement on whether measurements were taken from distinct samples or whether the same sample was measured repeatedly
☒	☐	The statistical test(s) used AND whether they are one- or two-sided<br>*Only common tests should be described solely by name; describe more complex techniques in the Methods section.*
☒	☐	A description of all covariates tested
☒	☐	A description of any assumptions or corrections, such as tests of normality and adjustment for multiple comparisons
☐	☒	A full description of the statistical parameters including central tendency (e.g. means) or other basic estimates (e.g. regression coefficient) AND variation (e.g. standard deviation) or associated estimates of uncertainty (e.g. confidence intervals)
☒	☐	For null hypothesis testing, the test statistic (e.g. F, t, r) with confidence intervals, effect sizes, degrees of freedom and P value noted<br>*Give P values as exact values whenever suitable.*
☒	☐	For Bayesian analysis, information on the choice of priors and Markov chain Monte Carlo settings
☒	☐	For hierarchical and complex designs, identification of the appropriate level for tests and full reporting of outcomes
☒	☐	Estimates of effect sizes (e.g. Cohen's d, Pearson's r), indicating how they were calculated

Our web collection on statistics for biologists contains articles on many of the points above.

Software and code

Policy information about availability of computer code

Data collection	LabView 2017 optical tweezers software
Data analysis	Matlab 2017 optical tweezers trace analysis software

For manuscripts utilizing custom algorithms or software that are central to the research but not yet described in published literature, software must be made available to editors and reviewers. We strongly encourage code deposition in a community repository (e.g. GitHub). See the Nature Portfolio guidelines for submitting code & software for further information.

Data

Policy information about availability of data

All manuscripts must include a data availability statement. This statement should provide the following information, where applicable:

- Accession codes, unique identifiers, or web links for publicly available datasets
- A description of any restrictions on data availability
- For clinical datasets or third party data, please ensure that the statement adheres to our policy

Relevant source data for main text and supplementary figures are provided in the Source Data section. All other data that support the findings of this study are available from the corresponding author upon reasonable request.

Field-specific reporting

Please select the one below that is the best fit for your research. If you are not sure, read the appropriate sections before making your selection.

☒ Life sciences ☐ Behavioural & social sciences ☐ Ecological, evolutionary & environmental sciences

For a reference copy of the document with all sections, see nature.com/documents/nr-reporting-summary-flat.pdf

Life sciences study design

All studies must disclose on these points even when the disclosure is negative.

Sample size	The sample size for each condition was 5-8 biologically independent sample chambers. Each sample chamber represents a unique assembly of proteins onto multiple DNA substrates. Note that within each sample chamber, multiple traces (on the order of 50 traces, see Source Data Files), each on a different DNA molecule, were measured to characterize the presence and location of proteins on the DNA. No sample size calculation was performed. The sample size was determined based on our experience with single-molecule measurements in order to accommodate variations in protein-DNA binding efficiency.
Data exclusions	No data were excluded from the analysis unless there were DNA tethering issues (e.g., DNA broke during measurement).
Replication	All the experiments described in the manuscript were reproducible.
Randomization	Randomization was not relevant to this study, as all experiments were carried out using the same experimental procedures.
Blinding	Blinding was not relevant to this study. All data collection was done following the same standard procedures.

Reporting for specific materials, systems and methods

We require information from authors about some types of materials, experimental systems and methods used in many studies. Here, indicate whether each material, system or method listed is relevant to your study. If you are not sure if a list item applies to your research, read the appropriate section before selecting a response.

Materials & experimental systems

n/a	Involved in the study	
☐	☒	Antibodies
☒	☐	Eukaryotic cell lines
☒	☐	Palaeontology and archaeology
☒	☐	Animals and other organisms
☒	☐	Human research participants
☒	☐	Clinical data
☒	☐	Dual use research of concern

Methods

n/a	Involved in the study	
☒	☐	ChIP-seq
☒	☐	Flow cytometry
☒	☐	MRI-based neuroimaging

Antibodies

Antibodies used	Goat anti-digoxygenin, Vector Labs, #MB-7000, lot ZD0727
Validation	Anti-digoxygenin was used to tether DNA to the surface. It was validated by the observation of DNA labeled with digoxygenin tethered to the surface. The specificity and quality of the anti-body does not affect the outcome of the single-molecule study. No additional data are necessary to validate the antibody.
