# Supplementary figures and images for: Polarity of the CRISPR roadblock to transcription
Source: Nat Struct Mol Biol. 2022 Dec 5;29(12):1217–27. doi: 10.1038/s41594-022-00864-x (PMC9758054; doi:10.1038/s41594-022-00864-x)

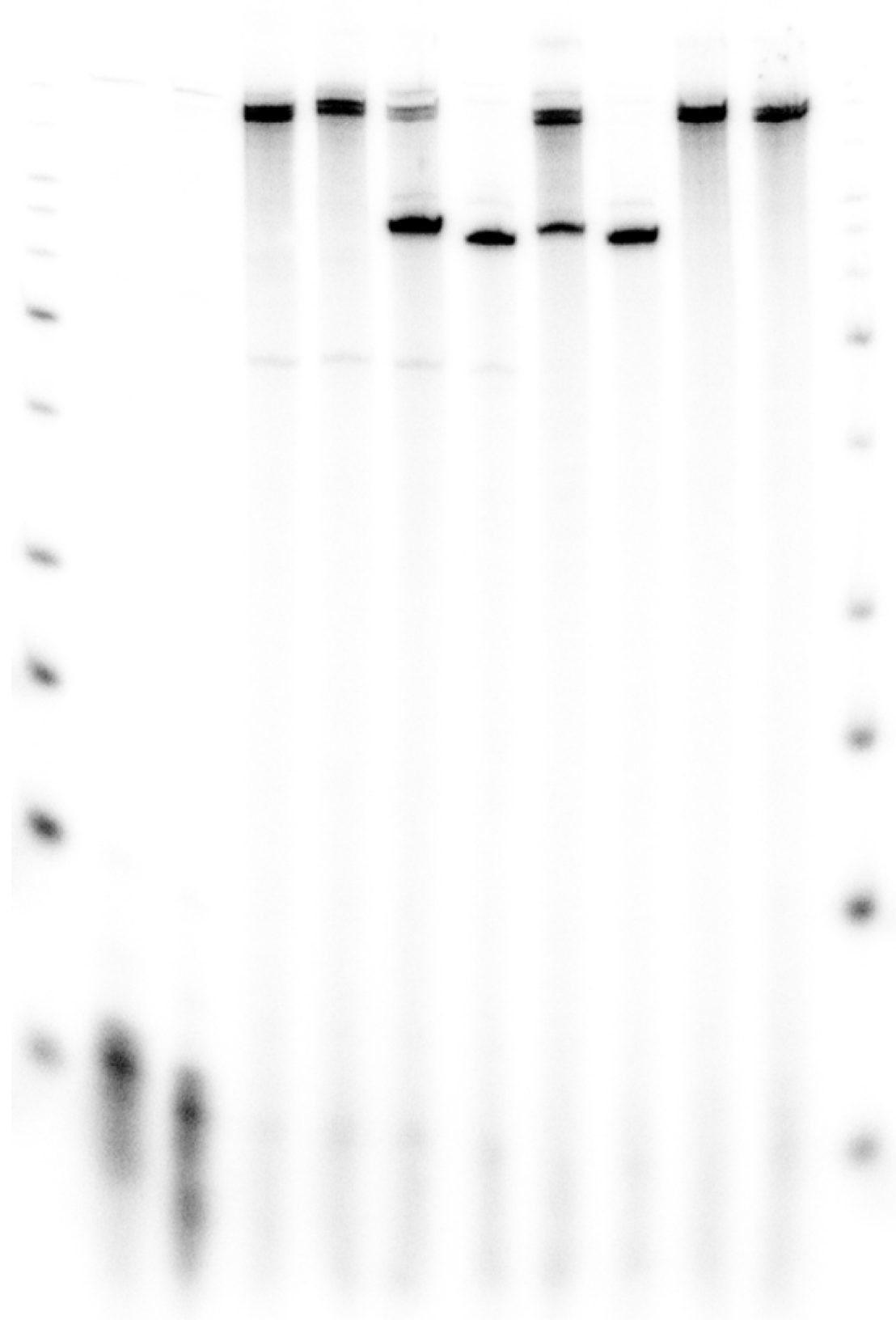

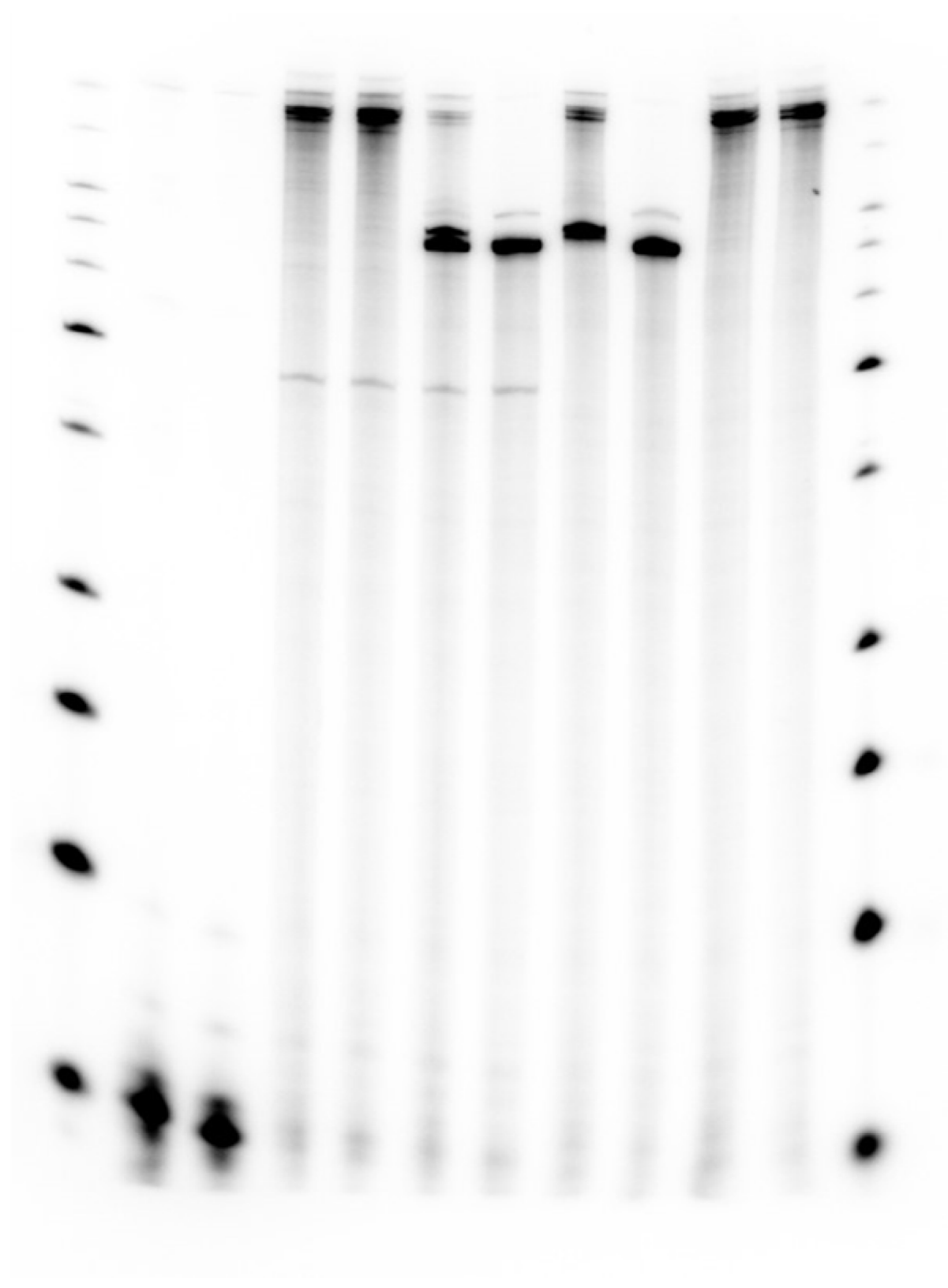

Supplement: Source Data Extended Data Fig. 6 — Uncropped gel images for a and b. [file 41594_2022_864_MOESM13_ESM.pdf]
